# OTOGL, a gelforming mucin protein, is nonessential for male germ cell development and spermatogenesis in mice

**DOI:** 10.1186/s12958-021-00779-0

**Published:** 2021-06-26

**Authors:** Zhiming Li, Yan Zhang, Xinzong Zhang, Congcong Cao, Xiaomin Luo, Yaoting Gui, Yunge Tang, Shuiqiao Yuan

**Affiliations:** 1 Institute of Reproductive Health, Tongji Medical College, Huazhong University of Science and Technology, 430030 Wuhan, Hubei China; 2 NHC Key Laboratory of Male Reproduction and Genetics, Family Planning Research Institute of Guangdong Province, Guangzhou, China; 3 Guangdong and Shenzhen Key Laboratory of Male Reproductive Medicine and Genetics, Institute of Urology, Peking University Shenzhen Hospital, Shenzhen Peking University- Hong Kong University of Science and Technology Medical Center, 518036 Shenzhen, China; 4 Shenzhen Huazhong University of Science and Technology Research Institute, Shenzhen, Guangdong China

**Keywords:** OTOGL, Spermatogenesis, Fertility, Knockout mouse model, CRISPR/Cas9

## Abstract

Otogelin-like protein (encoded by *Otogl*) was highly structural similar to the gelforming mucin proteins. Although human OTOG mutations have been linked to deafness, the biological function of OTOGL in male germ cell development remains enigmatic. In screening 336 patients with non-obstructive azoospermia (NOA), OTOGL displays the high mutant ratio (13.99 %). Then, we examined the expression of OTOGL in developing mouse testes. *Otogl* mRNA and protein are continually expressed in postnatal developing testes from postnatal day 0 (P0) testes to P21 testes exhibiting a decreased trend with the age growth. We thus generated a global *Otogl* knockout mouse (KO) model using the CRISPR/Cas9 technology; however, *Otogl* KO mice displayed normal development and fertility. Further histological analysis of *Otogl* knockout mouse testes revealed that all types of spermatogenic cells are present in *Otogl* KO seminiferous tubules. Together, our study suggested that OTOGL is nonessential for male germ cell development and spermatogenesis.

**Supplementary Information:**

The online version contains supplementary material available at 10.1186/s12958-021-00779-0.

## Introduction

Mucins are high molecular weight glycoproteins produced by many epithelial tissues. These include the membrane-associated mucins and the secreted mucins [1]. Membrane-associated mucins are expressed in various epithelial cells to form the glycocalyx at mucosal surfaces and act as a barrier to protect cells from infections. Secreted mucins serve as an anti-adhesion molecule and contribute to signal transduction. The expression of mucin isoforms has been reported in germ cells. Notably, Mucin 1(*Muc1*) showed the highest expression in mature spermatozoa, involved in sperm maturation and transportation along with the reproductive system [2]. Further study of mucins in the male reproductive tissues may yield information regarding the functional importance of mucins in male fertility.

Otogelin-like (OTOGL) is a type of gelforming mucin that forms high-molecular-weight complexes and is glycosylated through mucin-type glycosylation. OTOGL is a large protein with mucin-like domains. In 2017, Eamon Dubaiss et al. identified many proteins secreted from the epidermis of the *Xenopus tropicalis*, including an abundant protein called OTOGL, named because of its sequence similarity with a glycoprotein called Otogelin (OTOG) [3]. In humans, both OTOGL and OTOG were found in the acellular membranes of the inner ear and are associated with moderate autosomal recessive nonsyndromic hearing loss [4, 5]. Previously, OTOGL was considered as a novel candidate gene for 46, XY gonadal dysgenesis (GD) through a customized comparative genomic hybridization platform [6]. However, the functional role of OTOGL in the maintenance of normal male reproductive health was unknown.

In the current study, we found a high mutation ratio of OTOGLs in the patients with non-obstructive azoospermia (NOA) and *Otogl* mRNA highly expressed at the early stages of the testis tissues. To decipher the role of OTOGL in male germ cell development, we generated *Otogl* global knockout mouse (KO) model using CRISPR/Cas9 technology. However, *Otogl* KO male mice were fertile with normal testicular and epididymal histology. Our results showed that OTOGL is nonessential for male germ cell development and spermatogenesis in mice.

## Materials and methods

### Patients

Peripheral blood samples of 336 NOA patients used in this study were as previously described [7]. The inclusion criteria for the NOA patients included the following: (i) no sperm detected in the pellets of semen samples on three different occasions; (ii) no inflammation or injury of the reproductive system or pelvic cavity; and (iii) no karyotypic abnormality or Y chromosome microdeletion. Testicular biopsy and histological analysis were conducted for the azoospermic men whenever possible. This study was approved by the ethics committee of Peking University Shenzhen Hospital and Tongji Medical College in accordance with the Declaration of Helsinki (No. 20,090,018). Informed, written consents were obtained from all participants. .

### DNA extraction and sequencing

The selected exon sequencing and data analysis were performed, as described in detail previously [8]. Briefly, genomic DNA was extracted using the AllPrep DNA/RNA Mini Kit (Qiagen, Germantown, Maryland). The exon capture was performed using the NimbleGen custom array (Roche NimbleGen, Madison, Wisconsin). The sequencing (paired-end 90-base pair reads) was performed on an Illumina Hiseq 2000 platform (Illumina, San Diego, California) using recommended protocols from the manufacturer. After removing the low-quality bases and adaptor sequences, the sequencing reads were aligned against the human reference genome (NCBI build 37.1, hg19) using the SOAPaligner software.

### Mice

*Otogl* knockout mice were produced by zygote pronuclear microinjection using the CRISPR/Cas9 genome editing technique. The two pairs of single guided RNAs (sgRNAs) with the sequence sgRNA-1: TCAGTTAGGCTCCCTAATACTGG and sgRNA2: AATAGCTTACGTAACTTGCAAGG were designed for targeting from exon 9 to exon 23 of the *Otogl* gene. After microinjection, followed by Sanger sequencing confirmation and PCR analysis, the heterozygous with 669 bp deletion male founder mice were allowed to inbreed to produce homozygous mice. The mice were then housed in a specific pathogen-free facility under climate-controlled conditions with a 12-h light/dark cycle and were provided with water and a standard diet. All animal experiments have been specifically approved by the Institutional Animal Care and Use Committee (IACUC) of Tongji Medical College, Huazhong University of Science and Technology.

### Cell cultures and transfection

GC-2 cells (a mouse spermatocyte-derived cell line) were cultured in Dulbecco’s Modified Eagle Medium: Nutrient Mixture F-12 (DMEM/F-12) with phenol red supplemented with 10 % fetal bovine serum (FBS). The cells were incubated at 37 °C in a humidified atmosphere of 5 % CO2. pECMV-Otogl-m-Flag vector was purchased from Miaoling (Wuhan, China). The empty vector and pECMV-Otogl-mCherry-Flag were transfected into GC-2 cells by the standard lipofectamine 2000 procedure.

### RNA extraction and RT-qPCR

Trizol reagents (Invitrogen) were used for total RNA extraction, and reverse transcriptional reactions contained 500ng of purified total RNA using a PrimeScript RT reagent kit with gDNA Eraser (TaKaRa) to remove the DNA contamination. RT-qPCR was performed with SYBR green master mix (TaKaRa) on the ABI Step One System (Applied Biosystems) according to manufactures’ instructions: 95℃ for 10 min, 35 cycles of 95 °C for 1 min and 59 °C for 30 s. Otogl (forward: 5′-TCATTGGCTCTTGTTTCCTTG-3′; reverse: 5′-TTCTTCCGAGTCATCGTATTTT-3′). The primer sequence of spermatogenic genes were described in a previous study [9]. The relative gene expression was quantified using the comparative cycle threshold method, with the Gapdh expression used for normalization previously [10].

### Western blot

Testicular proteins were extracted using RIPA buffer. The protein lysates run on a 10 % SDS-PAGE gel and transferred to PVDF membranes. Membranes were blocked in 5 % non-fat milk (blocking solution) for 1 h and incubated with rabbit anti-OTOGL antibody (YT6398, Immnoway) at 4℃ for overnight. The membranes were washed with TBST three times, then incubated with HRP goat anti-rabbit IgG for 1 h. Excess reagents were washed three times in TBST and developed using chemiluminescence reagent was used for chemiluminescence detection and photographed by ChemiDoc XRS system (BIO-RAD).

### Histology analysis

For histological analysis, testes and epididymides were collected and fixed in Bouin’s fixative (Sigma Aldrich) for 3 h at room temperature. After several washes in 70 % ethanol to remove excess stain, testes were embedded in paraffin. Tissues were sectioned at 5 mm thickness and stained by Periodic Acid-Schiff (PAS)-hematoxylin.

### Immunofluorescence

Testes were fixed with 4 % paraformaldehyde (PFA) diluted with PBS and then embedded in the 50 % Tissue-Tek OCT compound (Sakura Finetek, 4583) in 20 % sucrose on liquid nitrogen. 5 μm cryosections were cut and treated with the antigen retrieval by 0.01 mM Citrate (PH = 6.0). The sections were washed three times in PBS (10 min/wash), then incubated with rabbit anti-DDX4 antibody (ab13840, Abcam) and rabbit anti-Ki67 antibody (ab15580, Abcam) in a humidified chamber at 4 ℃ for overnight. The sections were washed three times with PBS and then incubated with Alexa Fluor™ 594 Goat anti-rabbit IgG (Invitrogen, A11032) and PNA conjugated with fluorescein isothiocyanate at RT for 1 h. After washing three times with PBS (10 min per wash), the sections were counterstained with DAPI and photographed with the fluorescence microscope.

### Sperm counting

The cauda epididymis was dissected from adult mice. Sperm was squeezed out from the cauda epididymis and incubated in HTF medium for 30 min at 37 °C under 5 % CO_2_. Sperm counting analyses performed.

### Statistical analysis

The experimental data were analyzed and mapped by Graphad Prism5 software, and the mean-standard deviation was calculated. The Student’s *t-*test was used for inter-group differences. One-way ANNOVA was used for inter-group comparison of the number of births. *P* < 0.05 (*) was considered to have statistical significance.

## Results

### NOA patients carry high ratio of OTOGL mutations

Male infertility is one of the most serious problems facing modern society. A large number of gene mutations have been proposed as modulators of sperm production [11]. However, some of these genes have not been studied in Chinese NOA patients. To this end, we conducted sequence analysis of 13 disease-associated genes using next‐generation sequencing (NGS). Our study group consisted of 336 Chinese patients with NOA. The coding regions and exon‐intron boundaries of the target genes were analyzed by NGS‐based amplicon sequencing. Mutation screening was carried out for 13 probably associated genes with male infertility, namely, OTOGL [6], DOCK8 [12], GOLGA4[13], SALL1 [14], NRAP [15], SMYD4 [15], SLITRK1 [15], COBL [16], ZNF214 [17], LRFN2 [18], LRPPRC [19], OR2W3 [20], and CCDC77 [15]. Mutation analysis showed that 8 missense mutations of OTOGL were identified in 47 cases with NOA (13.99 %) (Table 1), suggesting that OTOGL may be the cause of NOA.

**Table 1:** Mutations found in the 336 NOA patients by the targeted NGS panel

Gene Name	mRNA ID	cDNA change	Mutation type	NOA hits in the tested cases*	Karyotype	AZF mutation
OTOGL	NM_001368062.3	c,412 C > T; c,901 A > G; c,1193 A > G; c,1440T > C; c,1678G > A; c,2155 C > G; c,2257G > A; c,2292G > A;	Missense	8 (*n* = 47)	46, XY	No
DOCK8	NM_203447.4	c,31G > A; c,37T > C; c,169 A > G; c,696 C > A; c,1024G > A; c,1284G > C; c,350 A > G	Missense	7(*n* = 45)	46, XY	No
GOLGA4	NM_002078.5	c,48T > C; c,411 A > C; c,452T > C; c,548T > C; c,686G > C; c,977G > A; c,1610T > C	Missense	7(*n* = 42)	46, XY	No
SALL1	NM_002968.3	c,360 A > C; c,588 A > G; c,676G > A; c,1071 C > T; c,1310 A > G	Missense	5(*n* = 18)	46, XY	No
NRAP	NM_006175.5	c,393 A > G; c,846T > C; c,1044 A > G; c,1435 C > G	Missense	4(*n* = 45)	46, XY	No
SMYD4	NM_052928.3	c,697T > C; c,557 C > T; c,331 C > T; c,221T > A	Missense	4(*n* = 17)	46, XY	No
SLITRK1	NM_052910.2	c,200T > G; c,295 C > T; c,650 C > T	Missense	3(*n* = 5)	46, XY	No
COBL	NM_015198.5	c,48 C > G; c,218 C > T; c,345 A > G	Missense	3(*n* = 16)	46, XY	No
ZNF214	NM_013249.4	c,390T > C; c,585T > C; c,410T > C	Missense	3(*n* = 12)	46, XY	No
LRFN2	NM_020737.3	c,266 A > C; c,572 A > C	Missense	2(*n* = 8)	46, XY	No
LRPPRC	NM_133259.4	c,1087 C > T; c,1196 A > C	Missense	2(*n* = 36)	46, XY	No
OR2W3	NM_001001957.2	c,142T > C; c,207 A > G	Missense	2(*n* = 13)	46, XY	No
CCDC77	NM_032358.4	c,48T > C; c,411 A > C	Missense	2(*n* = 11)	46, XY	No

*NOA* Non-obstructive azoospermia, *AZF* Azoospermia factor

### OTOGL highly expressed at the early stages of the mouse testes

**Fig. 1:**
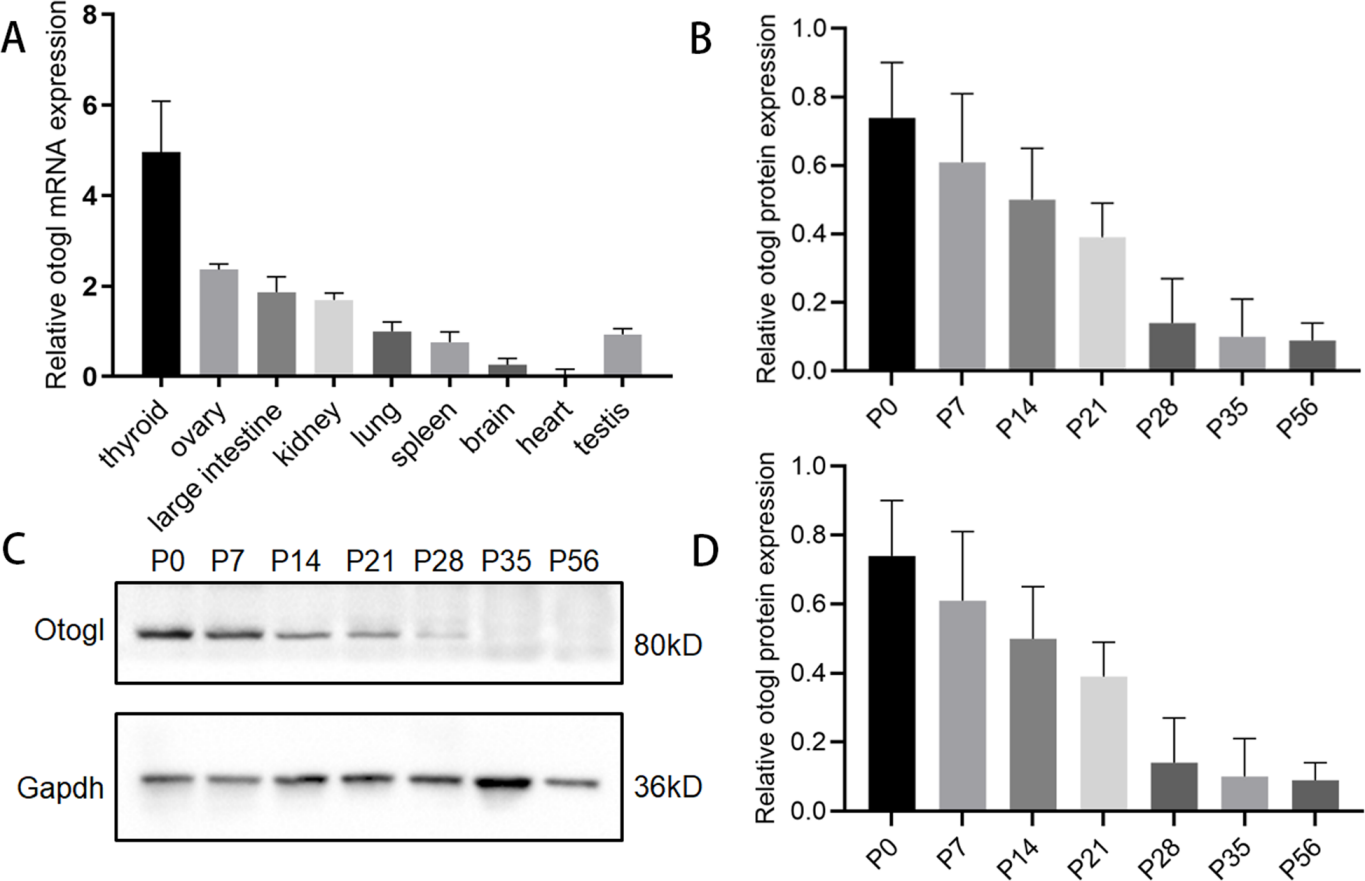
OTOGL is expressed in spermatogenic cells in mice. (**A**) RT-qPCR analyses of *Otogl* mRNA levels in nine organs of adult mice. (**B**) RT-qPCR analyses of *Otogl* mRNA levels in developing testes. Testes at postnatal Day 0 (P0), P7, P14, P21, P28, P35, and P56 were analyzed. *Gapdh* served as a loading control. (**C**) Western blotting shows the OTOGL protein levels in mouse testes at P0, P7, P14, P21, P28, P35, and P56. GAPDH served as a loading control. (**D**) Quantification analyses of OTOGL protein levels in developing testes at P0, P7, P14, P21, P28, P35, and P56. Data are presented as mean ± SD, *n* = 3

To determine the function of OTOGL in spermatogenesis and male germ cell development, we examined *Otogl* mRNA in multiple adult mouse tissues by RT-qPCR. We found that mRNA expression of *Otogl* is relatively low in the testis of the adult mice (Fig. 1A). We then examined the expression levels of *Otogl* in postnatal developing testes. The results showed that *Otogl* mRNA highly expressed in postnatal developing testes from postnatal day 0 (P0) testes to P21 testes, exhibiting its highest level at very early stages (Fig. 1B). Consistent with the mRNA expression, the protein levels of OTOGL displayed a gradual decrease from P7 to P56 (adulthood) (Fig. 1 C and 1D). Thus, we hypothesized that OTOGL might play an important role in early germ cell development and spermatogenesis.

### Generation of *Otogl* knockout mice

**Fig. 2:**
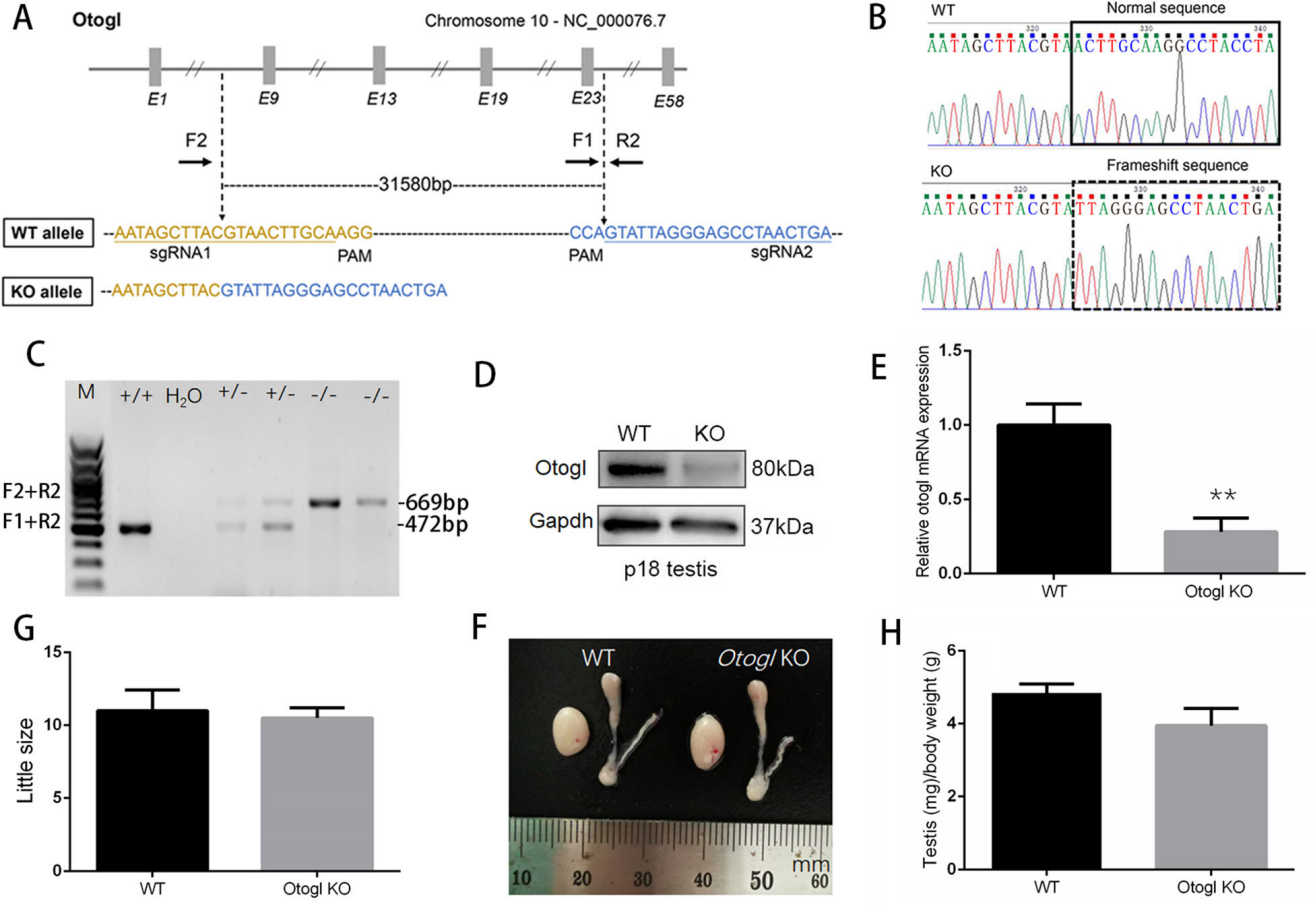
OTOGL is nonessential for spermatogenesis in mice. (**A**) Schematic illustration of the targeting strategy for generating OTOGL deficiency in mouse testes. (**B**) The sequence of WT and *Otogl* KO alleles are shown, respectively. (**C**) Representative PCR genotyping results show that KO bands (669 bp) were detected larger than WT bands (472 bp). (**D**) Representative Western blot analysis of OTOGL protein levels in WT and KO adult testes. GAPDH served as a loading control. (**E**) RT-qPCR assays showing elevated *Otogl* mRNA levels in KO adult testes. Data are presented as mean ± SD, n = 3. *P < 0.01 by student’s *t*-test. (**F**) Average litter size of pups produced by *Otogl* KO and WT males mated with WT females. Data are presented as mean ± SD, n = 3. (**G**) Similar gross morphology of WT and *Otogl* KO testes. One unit on the ruler is 1mm. (**H**) Testis/body weight ratio of WT and *Otogl* KO adult testes. Data are presented as mean ± SD, *n* = 3

To study the physiological role of OTOGL, we generated *Otogl* global knockout (KO) mice using CRISPR/Cas9 technology. Two sgRNAs were designed to target exons 9–23 for inactivation of the *Otogl* gene globally and verified by Sanger sequence (Fig. 2 A and B). The genotype of *Otogl* KO mice was confirmed by PCR-based genotyping analyses (Fig. 2 C). In addition to genotyping analyses, both mRNA and proteins of *Otogl* were appeared to be significantly reduced in *Otogl* KO testes compared with that of WT controls by Western blot and RT-qPCR (Fig. 2D and E). These results suggest that *Otogl* was inactivated in testes efficiently. Moreover, we found that *Otogl* KO mice were viable, and did not exhibit discernable differences in either growth or behavior compared to their wild-type (WT) or heterozygous littermates.

### Otogl is nonessential for spermatogenesis

**Fig. 3:**
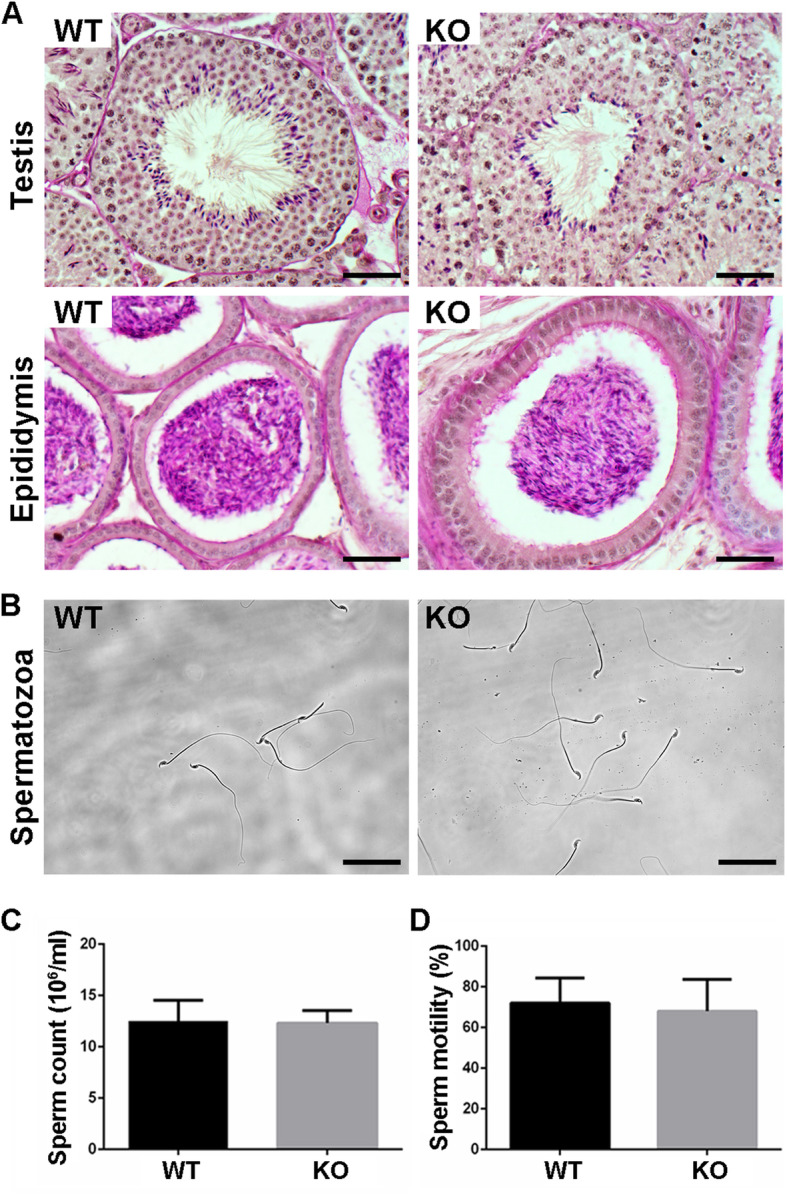
*Otogl* KO mice displayed normal testis histology and sperm morphology. (**A**) Periodic acid-Schiff (PAS) staining showing the histology of testis and epididymis (corpus) from adult WT and *Otogl* KO mice. Scale bar = 50 μm. (**B**) The representative phase-contrast micrograph is showing the normal morphology of *Otogl* KO spermatozoa. Scale bar = 50 μm. (**C**) Quantitative representation of sperm counts and (**D**) sperm motility from WT and *Otogl* KO mice. Data are presented as mean ± SD, *n* = 3

**Fig. 4:**
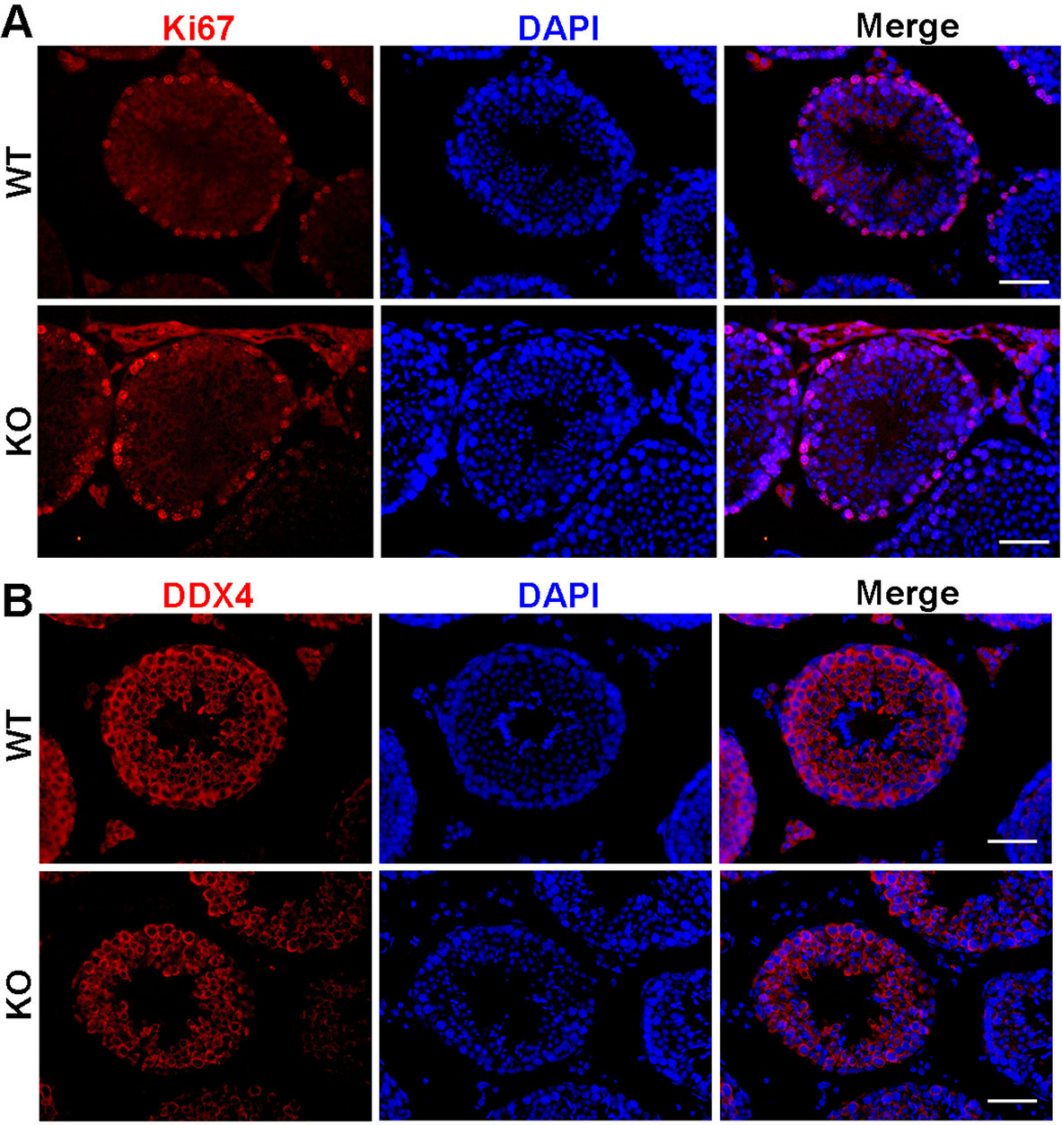
*Otogl* KO mice have normal germ cell proliferation and development. (**A**) No changes in the amounts of proliferating cells stained with Ki67 (red, a cell proliferative marker) were detected. Scale bar = 50 μm (**B**) Cellular localization of DDX4 (red, a germ cell marker) in WT and *Otogl* KO adult testes. Scale bar = 50 μm

To explore the role of OTOGL in spermatogenesis and male fertility, we performed a 5-month-long fecundity test using *Otogl* KO males bred with WT females of proven fertility. The result showed no significant difference in average litter size between WT and KO breeding pairs (Fig. 2 F), suggesting that *Otogl* KO males are fertile. Consistent with their normal fertility, testis size and weight of adult *Otogl* KO males were similar to those of WT males (Fig. 2G H). We then carried out Periodic acid-Schiff (PAS) staining to assess the morphology of testis and epididymis sections from WT and *Otogl* KO males. The results showed that the seminiferous tubules of *Otogl* KO mice are morphologically intact with normal spermatogenic cells at all stages from spermatogonia to spermatozoa (Fig. 3 A), suggesting that the entire process of spermatogenesis are not disturbed upon OTOGL depletion. Moreover, there are no detectable morphological differences of epididymides between WT and *Otogl* KO males (Fig. 3B). Consistent with these results, the number and motility of spermatozoa retrieved from cauda epididymis were comparable between WT and KO mice (Fig. 3 C and 3D), indicating that OTOGL deletion in mice does not affect the quality of spermatozoa. In addition, we stained frozen testis sections via Ki67 (a mitotically active cell marker) and DDX4 (a germ cell marker) by immunofluorescence. The results showed that the intensities of Ki67 and DDX4 were comparable between *Otogl* KO and WT testes (Fig. 4 A and 4B). Additionally, we overexpressed *Otogl* in GC-2 cell and did not find significant changes of spermatogenic genes in transcriptional level, including pluripotency factors (*Oct4* and *Nanog*), germ cell markers (*Stella, Mvh* and *Stra8*), haploid gamete markers (*Acrosin* and *Haprin*), and sperm cell makers (*Prm1* and *Prm2*) (Additional file 1: Figure S1). Taken together, our data suggested that OTOGL is nonessential for mouse spermatogenesis and male germ cell development.

## Discussion

Mucins play important barrier roles in reproductive processes and protection from bacterial pathogenesis in the female reproductive tract. However, little is known about the function of the mucin gene in spermatogenesis and male germ cell development. In this study, we focused on OTOGL because many mutations were identified from NOA patients via whole-exome sequencing. Previous study demonstrated that OTOGL was present in all three membranes of the mammalian inner ear, suggesting a fundamental and ancient role for this protein [4]. Unexpectedly, this study showed that OTOGL is not essential for spermatogenesis and male germ cell development in mice, although *Otogl* has a higher transcription level in the neonatal stages of the testis. High levels of transcription of *Otogl* in early testis development suggest that *Otogl* is normally involved in the production of early germ cells, which may require relatively low gene activity for maintenance continuously. Additionally, a recent study showed that otogelin-like, otogelin, and stereocilin form links connecting hair cell-stereocilia, required for normal hearing [21]. However, we found that, in the current study, OTOGL is not essential for male germ cell development and spermatogenesis using *in vivo* functional knockout mouse model, which suggests that OTOGL may have a distinct function in different organs or tissues. Of note, in this study, *Otogl* KO mice are generated and examined the phenotype were under normal conditions; we thus cannot exclude the possibility that *Otogl* KO mice might present abnormal spermatogenesis under the external stimulus. Therefore, the *Otogl* mutant mice will be useful for genetically testing the protective role of OTOGL under stress-inducible conditions in the future.

Previous study showed that some mutations, including c.6467 C > A [22], c.547 C > T [4, 23], c.6347 C > T [23], c.6559 C > T [23], in OTOGL are associated with autosomal recessive deafness. In current study we found some different mutations (c,412 C > T, c,901 A > G, c,1193 A > G, c,1440T > C, c,1678G > A, c,2155 C > G, c,2257G > A, and c,2292G > A) of OTOGL in NOA patients by NGS. Despite the fact that our loss of function study in mice suggested that this gene is not essential for normal spermatogenesis function, these mutations may impact OTOGL protein function in the other biological process, such as inner ear function. Interestingly, in mouse adult testis tissues, relatively low Otogl transcript levels were observed compared to fetal testis tissues. Consistent with these observations, *Otogl* mRNA levels were upregulated up to postnatal 13 days in the cochlea and downregulated in the adult cochlea [4]. Together, these results suggest that OTOGL may play a role in early organ development.

## Conclusions

In summary, we described the expression *Otogl* in mouse testes. Our *Otogl* KO mouse model showed that OTOGL is nonessential for male germ cell development and spermatogenesis.

## Supplementary Information

**Additional file 1: Figure S1.** The RT-qPCR analysis of the expression profile of spermatogenetic genes in GC-2 cells transfected with empty vector and pECMV-Otogl-mCherry-Flag in 48-hour post-transfection. Quantitate the expression of the following genes, including pluripotency factors (*Oct4* and *Nanog*), germ cell markers (*Stella*, *Mvh*, and *Stra8*), haploid gamete markers (*Acrosin* and *Haprin*), and sperm cell makers (*Prm1* and *Prm2*). All values were normalized to *Gapdh* (a housekeeping gene). Data are shown as mean ± SD, *n* = 3. Control was an empty vector transfection group. ns, not significant. ***, *P* < 0.001 by student’s *t*-test.

## Data Availability

Not applicable.

## Abbreviations

OTOGL: Otogelin-like
KO: Knockout
NOA: Non‐obstructive azoospermia
WT: Wild-type
PFA: Paraformaldehyde

## References

[1] Russo CL, Spurr-Michaud S, Tisdale A, Pudney J, Anderson D, Gipson IK (2006). Mucin gene expression in human male urogenital tract epithelia. Hum Reprod.

[2] Martinez-Conejero JA, Garrido N, Remohi J, Pellicer A, Simon C, Meseguer M (2008). MUC1 in human testis and ejaculated spermatozoa and its relationship to male fertility status. Fertil Steril.

[3] Dubaissi E, Rousseau K, Hughes GW, Ridley C, Grencis RK, Roberts IS, Thornton DJ (2018). Functional characterization of the mucus barrier on the Xenopus tropicalis skin surface. Proc Natl Acad Sci U S A.

[4] Yariz KO, Duman D, Zazo Seco C, Dallman J, Huang M, Peters TA, Sirmaci A, Lu N, Schraders M, Skromne I (2012). Mutations in OTOGL, encoding the inner ear protein otogelin-like, cause moderate sensorineural hearing loss. Am J Hum Genet.

[5] Avan P, Le Gal S, Michel V, Dupont T, Hardelin J-P, Petit C, Verpy E (2019). Otogelin, otogelin-like, and stereocilin form links connecting outer hair cell stereocilia to each other and the tectorial membrane. Proc Natl Acad Sci.

[6] Norling A, Hirschberg AL, Iwarsson E, Persson B, Wedell A, Barbaro M (2013). Novel candidate genes for 46, XY gonadal dysgenesis identified by a customized 1 M array-CGH platform. European Journal of Medical Genetics.

[7] Li Z, Huang Y, Li H, Hu J, Liu X, Jiang T, Sun G, Tang A, Sun X, Qian W (2015). Excess of rare variants in genes that are key epigenetic regulators of spermatogenesis in the patients with non-obstructive azoospermia. Sci Rep.

[8] Ma Q, Li Y, Guo H, Li C, Chen J, Luo M, Jiang Z, Li H, Gui Y (2016). A Novel Missense Mutation in USP26 Gene Is Associated With Nonobstructive Azoospermia. Reprod Sci.

[9] West JA, Park IH, Daley GQ, Geijsen N (2006). In vitro generation of germ cells from murine embryonic stem cells. Nat Protoc.

[10] Li Z, Zheng Z, Ruan J, Li Z, Zhuang X, Tzeng CM (2016). Integrated analysis miRNA and mRNA profiling in patients with severe oligozoospermia reveals miR-34c-3p downregulates PLCXD3 expression. Oncotarget.

[11] Nakamura S, Miyado M, Saito K, Katsumi M, Nakamura A, Kobori Y, Tanaka Y, Ishikawa H, Yoshida A, Okada H (2017). Next-generation sequencing for patients with non-obstructive azoospermia: implications for significant roles of monogenic/oligogenic mutations. Andrology.

[12] Lopes AM, Aston KI, Thompson E, Carvalho F, Goncalves J, Huang N, Matthiesen R, Noordam MJ, Quintela I, Ramu A (2013). Human spermatogenic failure purges deleterious mutation load from the autosomes and both sex chromosomes, including the gene DMRT1. PLoS Genet.

[13] Guo SS, Lv CY, Ouyang SJ, Wang XL, Liao AH, Yuan SQ (2020). GOLGA4, A Golgi matrix protein, is dispensable for spermatogenesis and male fertility in mice. Biochem Biophys Res Commun.

[14] Kohlhase J, Liebers M, Backe J, Baumann-Muller A, Bembea M, Destree A, Gattas M, Grussner S, Muller T, Mortier G (2003). High incidence of the R276X SALL1 mutation in sporadic but not familial Townes-Brocks syndrome and report of the first familial case. J Med Genet.

[15] Aston KI, Krausz C, Laface I, Ruiz-Castane E, Carrell DT (2010). Evaluation of 172 candidate polymorphisms for association with oligozoospermia or azoospermia in a large cohort of men of European descent. Hum Reprod.

[16] Kovac JR, Pastuszak AW, Lamb DJ (2013). The use of genomics, proteomics, and metabolomics in identifying biomarkers of male infertility. Fertil Steril.

[17] Gianotten J, van der Veen F, Alders M, Leschot NJ, Tanck MW, Land JA, Kremer JA, Hoefsloot LH, Mannens MM, Lombardi MP, Hoffer MJ (2003). Chromosomal region 11p15 is associated with male factor subfertility. Mol Hum Reprod.

[18] Aston KI, Carrell DT (2009). Genome-wide study of single-nucleotide polymorphisms associated with azoospermia and severe oligozoospermia. J Androl.

[19] Sasarman F, Nishimura T, Antonicka H, Weraarpachai W, Shoubridge EA, Consortium L (2015). Tissue-specific responses to the LRPPRC founder mutation in French Canadian Leigh Syndrome. Hum Mol Genet.

[20] Plaseski T, Noveski P, Popeska Z, Efremov GD, Plaseska-Karanfilska D (2012). Association study of single-nucleotide polymorphisms in FASLG, JMJDIA, LOC203413, TEX15, BRDT, OR2W3, INSR, and TAS2R38 genes with male infertility. J Androl.

[21] Avan P, Le Gal S, Michel V, Dupont T, Hardelin JP, Petit C, Verpy E (2019). Otogelin, otogelin-like, and stereocilin form links connecting outer hair cell stereocilia to each other and the tectorial membrane. Proc Natl Acad Sci U S A.

[22] Gu X, Sun S, Guo L, Lu X, Mei H, Lai C, Li H (2015). Novel biallelic OTOGL mutations in a Chinese family with moderate non-syndromic sensorineural hearing loss. Int J Pediatr Otorhinolaryngol.

[23] Oonk AM, Leijendeckers JM, Huygen PL, Schraders M, del Campo M, del Castillo I, Tekin M, Feenstra I, Beynon AJ, Kunst HP (2014). Similar phenotypes caused by mutations in OTOG and OTOGL. Ear Hear.

