# Correction to: OTOGL, a gelforming mucin protein, is nonessential for male germ cell development and spermatogenesis in mice

**DOI:** 10.1186/s12958-021-00803-3

**Published:** 2021-09-08

**Authors:** Zhiming Li, Yan Zhang, Xinzong Zhang, Congcong Cao, Xiaomin Luo, Yaoting Gui, Yunge Tang, Shuiqiao Yuan

**Affiliations:** 1 Institute of Reproductive Health, Tongji Medical College, Huazhong University of Science and Technology, Wuhan, 430030 Hubei China; 2 NHC Key Laboratory of Male Reproduction and Genetics, Family Planning Research Institute of Guangdong Province, Guangzhou, China; 3 Guangdong and Shenzhen Key Laboratory of Male Reproductive Medicine and Genetics, Institute of Urology, Peking University Shenzhen Hospital, Shenzhen Peking University- Hong Kong University of Science and Technology Medical Center, Shenzhen, 518036 China; 4 Shenzhen Huazhong University of Science and Technology Research Institute, Shenzhen, Guangdong China

**Correction to: Reprod Biol Endocrinol 19, 95 (2021)**

**https://doi.org/10.1186/s12958-021-00779-0**

Following publication of the original article [1], the authors reported an error in Fig. 1. The published Fig. 1B is an image of mRNA RT-qPCR analysis of Otogl levels in developing testes, rather than a protein quantification analysis image as labeled. We have attached a corrected version of Fig. 1B.

The labels of F and G in the published Fig. 2 should be interchanged. This error does not change the scientific conclusions of the article in any way. The authors apologize for this error.

The correct figures are presented below.

**Fig. 1:**
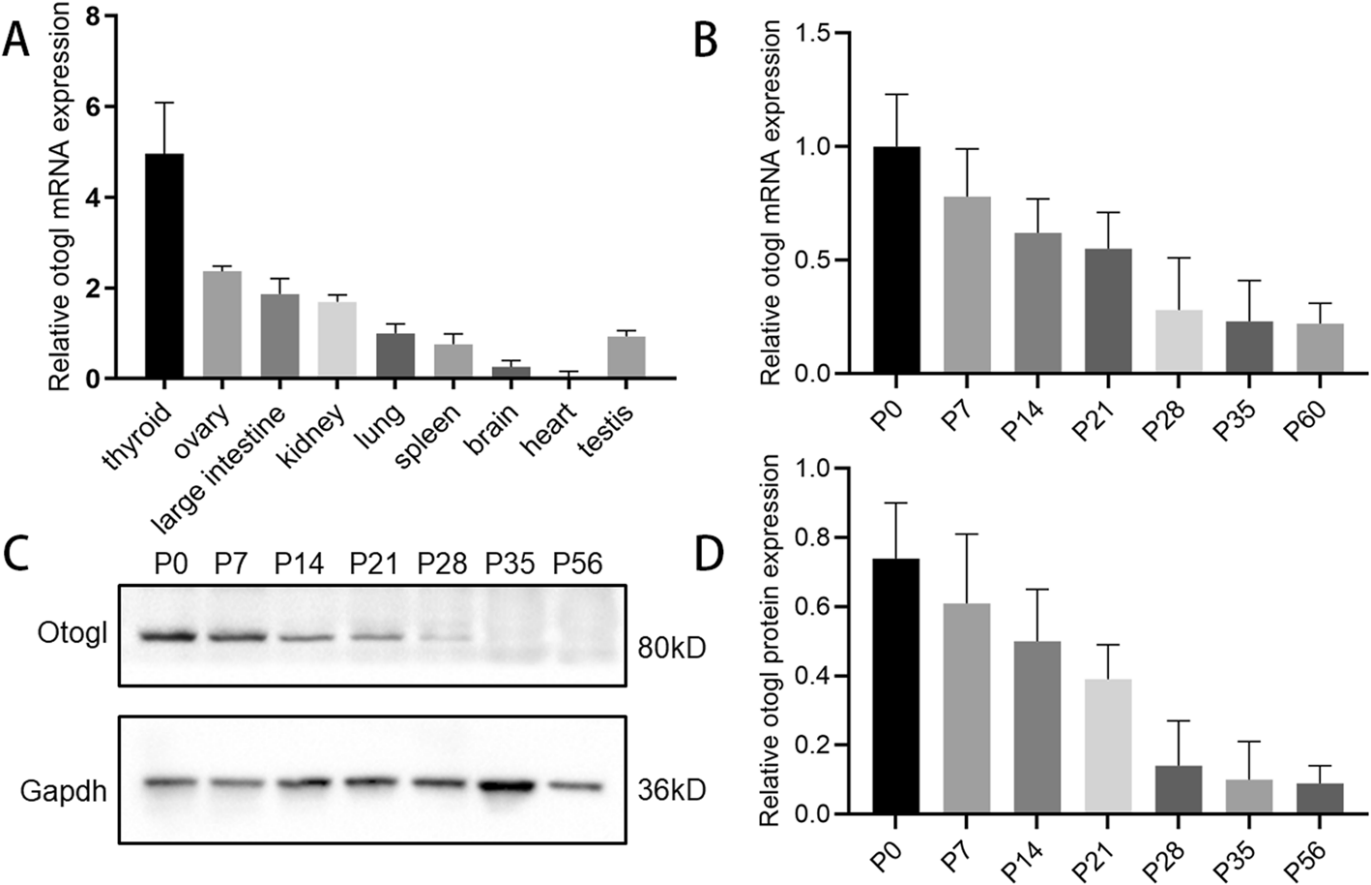
OTOGL is expressed in spermatogenic cells in mice. **A** RT-qPCR analyses of *Otogl* mRNA levels in nine organs of adult mice. **B** RT-qPCR analyses of *Otogl* mRNA levels in developing testes. Testes at postnatal Day 0 (P0), P7, P14, P21, P28, P35, and P56 were analyzed. *Gapdh* served as a loading control. **C** Western blotting shows the OTOGL protein levels in mouse testes at P0, P7, P14, P21, P28, P35, and P56. GAPDH served as a loading control. **D** Quantification analyses of OTOGL protein levels in developing testes at P0, P7, P14, P21, P28, P35, and P56. Data are presented as mean ± SD, *n* = 3

**Fig. 2:**
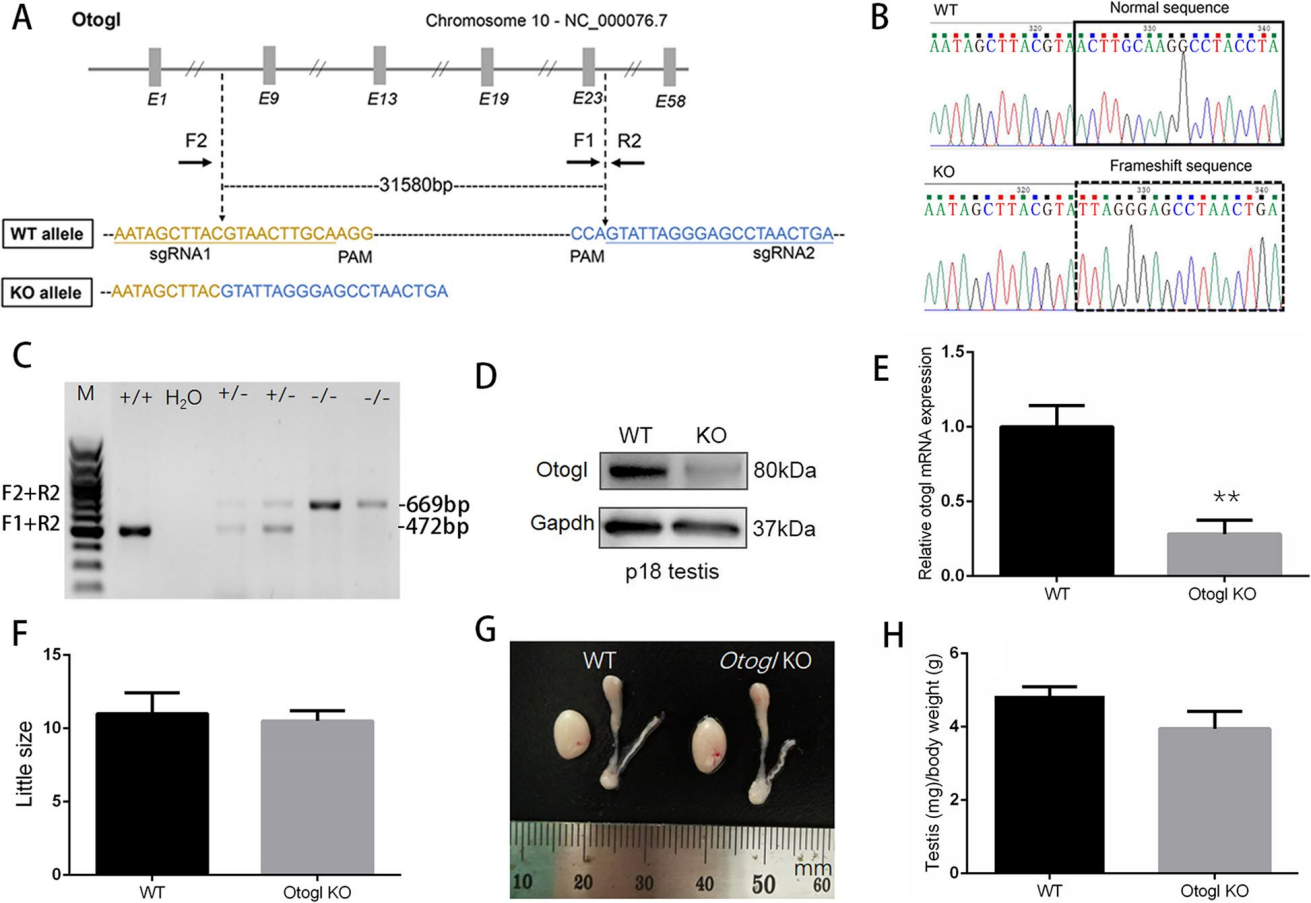
OTOGL is nonessential for spermatogenesis in mice. **A** Schematic illustration of the targeting strategy for generating OTOGL deficiency in mouse testes. **B** The sequence of WT and *Otogl* KO alleles are shown, respectively. **C** Representative PCR genotyping results show that KO bands (669 bp) were detected larger than WT bands (472 bp). **D** Representative Western blot analysis of OTOGL protein levels in WT and KO adult testes. GAPDH served as a loading control. **E** RT-qPCR assays showing elevated *Otogl* mRNA levels in KO adult testes. Data are presented as mean ± SD, *n* = 3. *P < 0.01 by student’s t-test. **F** Average litter size of pups produced by *Otogl* KO and WT males mated with WT females. Data are presented as mean ± SD, *n* = 3. **G** Similar gross morphology of WT and *Otogl* KO testes. One unit on the ruler is 1mm. **H** Testis/body weight ratio of WT and *Otogl* KO adult testes. Data are presented as mean ± SD, *n* = 3

The original article [1] has been updated.
