# Supplementary material for: OTOGL, a gelforming mucin protein, is nonessential for male germ cell development and spermatogenesis in mice
Source: Reprod Biol Endocrinol. 2021 Jun 26;19:95. doi: 10.1186/s12958-021-00779-0 (PMC8234668; doi:10.1186/s12958-021-00779-0)
Supplement: Supplementary file 1 — Additional file 1: Figure S1. The RT-qPCR analysis of the expression profile of spermatogenetic genes in GC-2 cells transfected with empty vector and pECMV-Otogl-mCherry-Flag in 48-hour post-transfection. Quantitate the expression of the following genes, including pluripotency factors (Oct4 and Nanog), germ cell markers (Stella, Mvh, and Stra8), haploid gamete markers (Acrosin and Haprin), and sperm cell makers (Prm1 and Prm2). All values were normalized to Gapdh (a housekeeping gene). Data are shown as mean ± SD, n = 3. Control was an empty vector transfection group. ns, not significant. ***, P < 0.001 by student’s t-test. [file 12958_2021_779_MOESM1_ESM.docx]

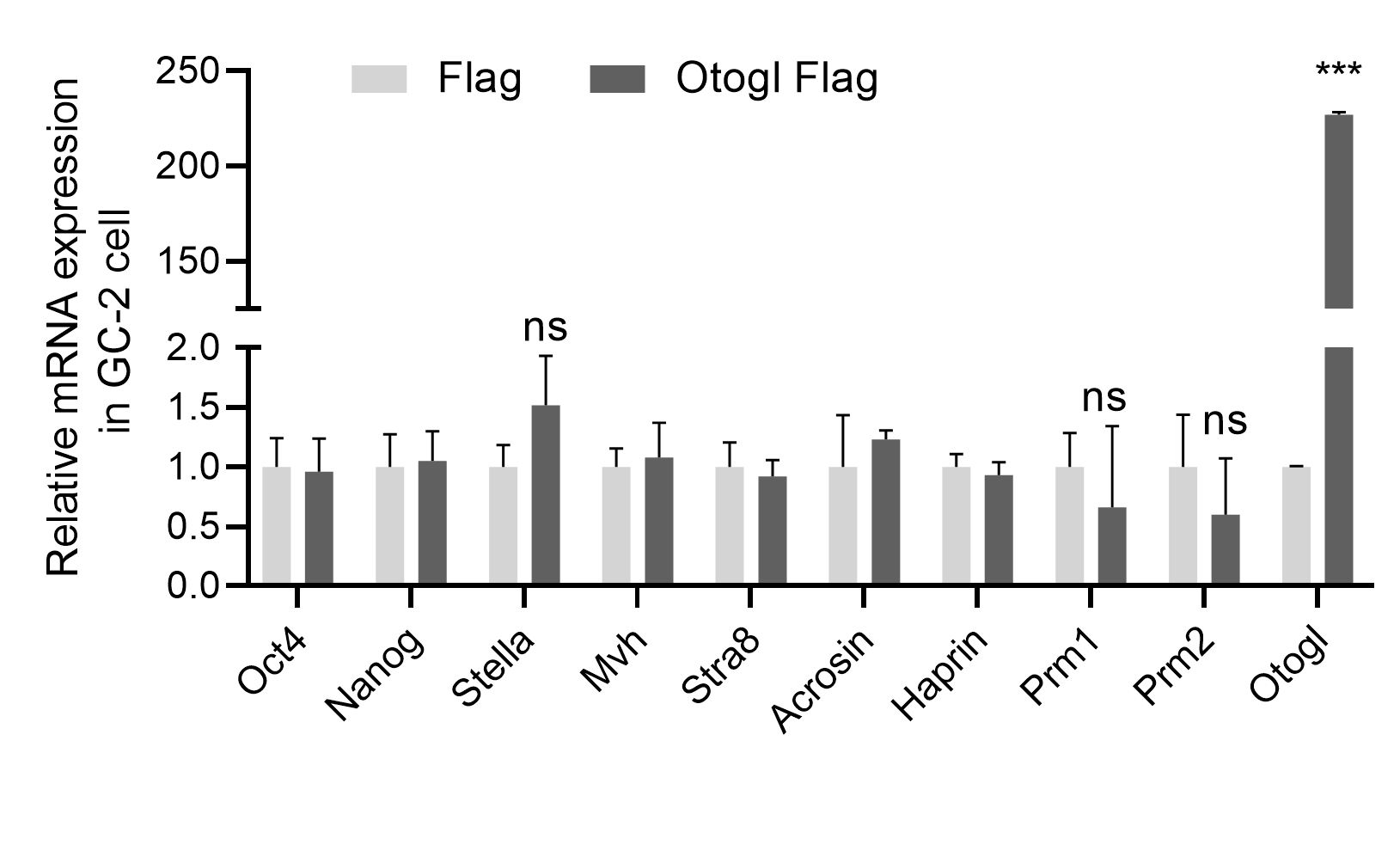


Figure S1. The RT-qPCR analysis of the expression profile of spermatogenetic genes in GC-2 cells transfected with empty vector and pECMV-Otogl-mCherry-Flag in 48-hour post-transfection. Quantitate the expression of the following genes, including pluripotency factors (*Oct4* and *Nanog*), germ cell markers (*Stella*, *Mvh*, and *Stra8*), haploid gamete markers (*Acrosin* and *Haprin*), and sperm cell makers (*Prm1* and *Prm2*). All values were normalized to *Gapdh* (a housekeeping gene). Data are shown as mean ± SD, n = 3. Control was an empty vector transfection group. ns, not signiﬁcant. ***, *P* <0.001 by student’s *t-*test.
